# Optimizing Detection of Circulating Tumor Cells in Breast Cancer: Unveiling New Markers for Clinical Applications

**DOI:** 10.3390/ijms26104714

**Published:** 2025-05-14

**Authors:** Amira Mehtar, Janine Wechsler, Christophe Desterke, Julien Giron-Michel, Amira Bouzidi, Aude Burlion, Fawzia Louache, Samira Kahia-Tani, Georges Uzan, Sina Naserian

**Affiliations:** 1 ScreenCell, 75012 Paris, France; amehtar@screencell.com (A.M.); jwechsler@screencell.com (J.W.); abouzidi@screencell.com (A.B.); aburlion@screencell.com (A.B.); 2 Faculté de Médecine du Kremlin Bicêtre, Université Paris-Saclay, INSERM UMRS-1310, 94805 Villejuif, France; christophe.desterke@inserm.fr; 3 Orsay-Vallée Campus, Paris-Saclay University, 91190 Gif-sur-Yvette, France; julien.giron-michel@inserm.fr (J.G.-M.); fawzia.louache@inserm.fr (F.L.); georges.uzan@inserm.fr (G.U.); 4 Inserm UMR-S-MD 1197, Paul-Brousse Hospital, 94800 Villejuif, France; 5 Kahia Laboratory, Oran 31000, Algeria; dr.samira@kahiadiagnostic.com; 6 Genethical, Oran 31000, Algeria

**Keywords:** liquid biopsy, precision medicine, circulating tumor cells, breast cancer, biomarker

## Abstract

Breast cancer (BC) is a heterogeneous disease with high metastasis potential, especially in the bones, liver, and lungs. Circulating tumor cells (CTCs), which emerge from active tumors, represent an early step toward metastasis and are associated with poor prognosis. CTCs of carcinoma origin are believed to express EpCAM and cytokeratins (CKs), common epithelial markers that are frequently used to identify them. However, in practice, the most aggressive CTCs lose the expression of those markers, leading to the partial loss of important information. Thus, finding some novel markers that identify CTCs regardless of their heterogeneity is crucial. A specific bioinformatics workflow integrating primary tumor and diverse BC cell lines transcriptomic expression analysis was developed and compared with single CTC transcriptomic analyses. We have identified a set of genes that are overexpressed in primary BC cells and are commonly upregulated among BC cell lines. Fifty of them were also found to be expressed in BC CTCs by single-cell transcriptomic analysis. Further in silico sorting narrowed this list to 12 genes. Using ScreenCell technology to isolate cancer cells spiked into normal blood, we tested the protein expression of all corresponding genes in vitro using the double immunocytochemistry method and validated MARCKSL1, SLC9A3R1, and RHOD as the most expressed markers. We then isolated the CTCs of 40 LN-invaded BC patients and 18 healthy donors using ScreenCell technology and showed that the combination of these three markers resulted in significantly better recognition of CTCs compared to EpCAM and CK conventional markers. Employing these novel markers, we found a clear distinction between blood samples from patients and healthy donors. In conclusion, through a specific bioinformatics workflow, in addition to in vitro and further clinical validations, we found three novel markers to precisely identify CTCs. These markers, when used together, enable a significantly more efficient identification of CTCs compared to conventional epithelial markers.

## 1. Background

According to the World Health Organization (WHO), breast cancer (BC) is the most common malignant neoplasm in women and is one of the leading causes of cancer deaths, especially in its metastatic stage.

Metastasis is a complex process that causes cancer cells to acquire different new features, such as increased mobility, invasion capabilities, and resistance to cell death at specific moments, like during tissue invasion and when establishing secondary tumors. Circulating tumor cells (CTCs) are in an intermediate step of the entire metastatic process. Indeed, it is well accepted that metastasis is predominantly mediated by blood circulation and lymphatic transmission of CTCs that arise from primary tumors and seed into distant organs for secondary tumor growth [1,2,3]. However, it is still unclear when a tumor’s metastatic potential emerges. Direct and indirect data contradict the hypothesis that tumor cells spreading to secondary sites is a late occurrence in carcinogenesis [4]. For instance, micrometastases in the bone marrow are seen in 30.6% of BC patients upon diagnosis, regardless of disease stage [5]. Therefore, the evaluation of CTCs could provide crucial information at all cancer stages.

Iterative tissue biopsies are invasive and not always possible to perform [6]. Evaluation of CTCs isolated from peripheral blood as a “liquid biopsy” is an appealing option, allowing real-time cancer follow-up regardless of tumor heterogeneity. BC CTCs exist in variable sizes [7] as single cells or cluster forms, with the latter having significantly more metastatic ability and a worse prognosis [8]. Studies have shown that most CTCs develop anoikis or are damaged due to the shear stress in blood circulation. Only a small fraction of CTCs can survive, and even a smaller population of them displays high metastatic potential [9]. Several possible CTC survival mechanisms have been demonstrated. Among them, genetic alterations, abnormal gene expression, epithelial-mesenchymal transition (EMT), stem cell properties, shielding effects of platelets, and, most importantly, immune escape mechanisms are shown to be of crucial importance [10,11]. Upregulation of different immunosuppressive molecules, such as programmed death-ligand 1 (PDL1) [12,13] and HLA-G [14] was shown to be among the most important mechanisms of BC CTCs’ immune escape. Despite significant progress in the development of single-cell approaches for the cellular and molecular characterization of CTCs, data on the functional properties of CTCs is still limited [15,16]. At early BC stages (non-metastatic stages I to III), CTC analysis was reported to be an independent predictor of poor disease-free, distant disease-free, and overall survival (OS) [17]. CTC analysis was also shown to be an indicator of treatment effectiveness, as the lower number of CTCs after treatment was associated with better relapse-free and OS [18,19,20]. In the metastatic stage of BC, a pooled analysis of 1944 patients demonstrated that a baseline CTC count of ≥5 per 7.5 mL was associated with decreased progression-free survival (PFS) and OS [21]. Another study reported that the CTC follow-up during treatment was a better predictor of PFS and OS compared to baseline CTC evaluation [22]. These clinical data shed light on the importance of the correct identification of CTCs.

CTCs of carcinoma origin are expected to express epithelial-specific markers such as EpCAM and/or cytokeratins (CK), which are not expressed in leukocytes [23]. Many CTC detection technologies are therefore developed based on EpCAM/CK recognition. However, several studies have evidenced a heterogeneous expression of these markers in CTCs. Notably, as a mechanism to improve CTC migratory and invasive properties, they undergo EMT, leading to significant downregulation of common epithelial markers’ expression [24,25,26,27]. Despite these limitations, EpCAM and CK markers are still used as conventional CTC isolation and characterization markers, although enrichment and immunostaining based on these two markers do not permit the recognition of all existing CTCs, leading to a partial loss of crucial information [28].

To address the CTC identification issue, ScreenCell has developed an antigen-independent and sensitive technology that enriches CTCs based on their bigger size compared to normal blood cells [29,30,31]. Once potential CTCs (according to morphological criteria) are efficiently isolated, immunostaining methods such as immunocytochemistry (ICC) or immunofluorescence (IF) and molecular biology methods such as digital PCR (dPCR) and next-generation sequencing (NGS) can be used to characterize those cells and confirm their tumorigenic nature [4,32].

The present study aimed to find novel and reliable markers that identify CTCs more efficiently and consistently. We focused our study on BC because of its high incidence. Also, it is the most frequently studied CTC model. We used bioinformatics to identify antigenic targets that are overexpressed on the plasma membrane of tumor cells using three distinct datasets: (1) primary BC cells, (2) the most common BC cell lines, and (3) BC CTCs.

The protein expression of the genes that were identified by the in silico investigations was evaluated in vitro using two BC cell lines (MCF7 and SKBR3) that were spiked into healthy blood samples. ScreenCell Cyto technology was used to isolate those tumor cells, and the expression of target proteins was verified using the ICC method. Three markers with the highest and the most specific expression were selected to be further evaluated, individually or combined, in a cohort of 40 lymph node (LN)-invaded BC patients and 18 healthy donors. Finally, CTCs from patients’ blood samples were isolated by ScreenCell Cyto technology, and the expression of these three new markers was compared with EpCAM and CK conventional markers.

## 2. Results

### 2.1. In Silico Investigations for Finding Novel Markers

**Figure 1:**
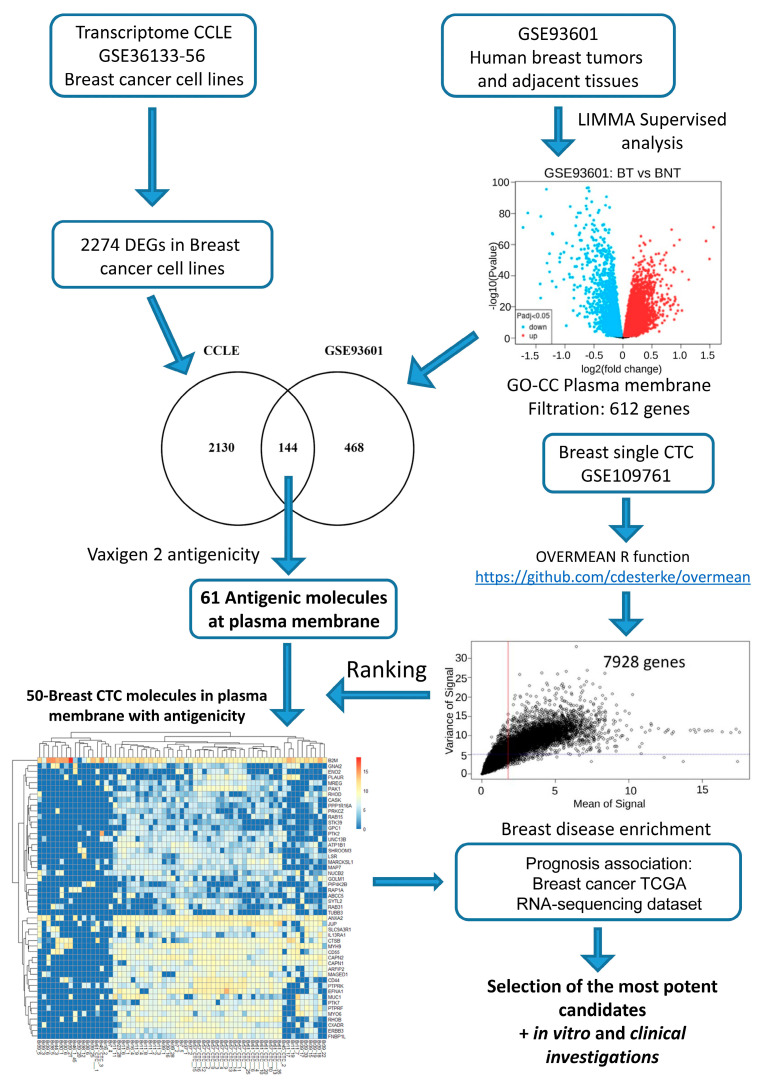
Workflow of the bioinformatics analyses. We observed that 114 genes were expressed in the diversity of CCLE BC cell lines and overexpressed on the plasma membrane of human breast tumor cells. Among them, 61 molecules were classed as antigenic by the Vaxijen2 algorithm. Finally, 50 of them were verified to be expressed in BC CTCs according to the single-cell transcriptome analysis.

We have established a specific bioinformatics workflow for BC transcriptome analysis (Figure 1). Firstly, we have explored overexpressed primary breast tumor markers for their heterogeneity of expression in BC cell lines, which led to the identification of 2274 genes. In parallel, a transcriptome-supervised analysis was used to identify 612 genes that are upregulated in primary breast tumors in comparison to breast-adjacent tissue samples and known to be present on the plasma membrane by querying the Gene Ontology Cell Compartment database. Secondly, in following downstream analysis, this plasma membrane molecule signature was merged with the first gene profile found across BC cell lines to find overlapping molecules. This process led to the identification of 144 commonly upregulated genes. Thirdly, BC’s differentially expressed gene (DEG) profile was restricted to antigenic molecules, evaluating the antigenicity of their sequence using the VaxiJen score bioinformatics tool (VaxiJen version 2.0) [33]. This process kept 61 out of 144 genes that were initially identified.

Finally, the expression of common BC antigenic markers was compared to that of BC CTCs. A complete single-cell transcriptome from a unique CTC dataset (GSE109761) was analyzed with the “overmean” R-function. This analysis revealed that BC CTCs expressed 7928 DEGs. Merging single-cell transcriptome analysis of CTCs with 61 overexpressed antigenic molecules in primary breast tumors demonstrated 50 of them being expressed in CTCs (Figure 1).

### 2.2. In Silico Filtration to Achieve a Final Blueprint of Potential Markers of Interest

We then performed a series of complementary in silico filtrations to preserve only the most relevant identified targets (Supplementary Figure S1). Therefore, we first made sure that the selected 50 genes were accurately expressed at the cell cytoplasmic membrane. To do this, we used two frequently used databases, i.e., Protein Atlas and UniProt. Data showed that 26 out of 50 genes have membrane expression according to both databases, and 4 out of 50 have membrane expression according to only one of those databases. All 30 genes were kept so as not to neglect any potential candidates. After blood filtration with ScreenCell technology, CTCs are retained on the IS along with a small fraction of large leukocytes. Thus, we eliminated the genes that are expressed by immune cells, verified by those two databases, to have the maximum CTC specificity, a process that led to the preservation of 19 target genes.

In the last step, we only kept 12 genes that, according to the online databases (The Human Protein Atlas), had an intermediate or high expression level (performed by IHC) in several primary tumor samples of BC patients (Supplementary Figure S1A). RNA sequencing data of different BC cell lines (Protein Atlas) enabled us to inspect which BC cell lines express those 12 genes. MCF7 (HR+ HER2−) and SKBR3 (HR− HER2+) cell lines were found to express all those genes of interest (Supplementary Figure S1B and Table 1).

### 2.3. Investigations on BC Cell Lines Revealed the Targets with the Most Specific and Highest Expression

**Figure 2:**
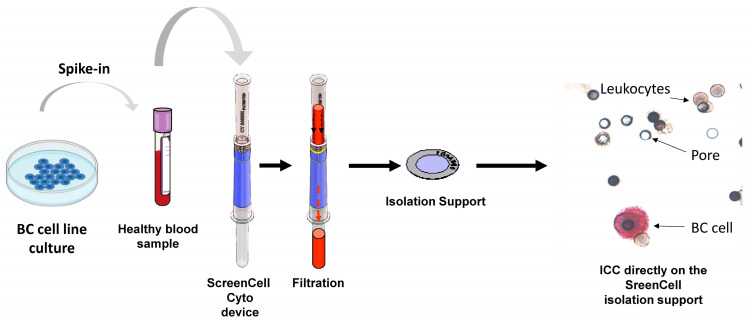
ScreenCell technology workflow. BC cell lines (MCF7 and SKBR3) were expanded in culture, and then 5000 cells were spiked into 3 mL of healthy blood samples. ScreenCell Cyto kits were then used to filter the blood to isolate cancer cells according to their bigger size. The ICC method was then used to stain leukocytes in brown (DAB) and BC cells in red (RED).

**Figure 3:**
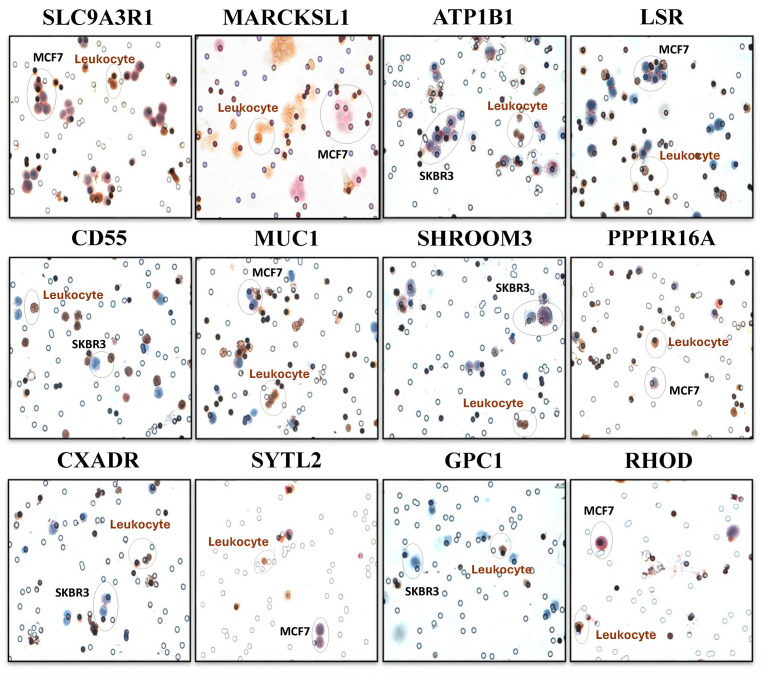
Representative images of ICC staining targeting each of the 12 proteins of interest. Leukocytes are stained using anti-CD45 antibody in brown, and cancer cells (MCF7 or SKBR3) are stained in red using each marker of interest. The cell nucleus is stained with hematoxylin. Images are captured at 40×.

After in silico studies, we performed in vitro experiments to validate the bioinformatics results. Therefore, we spiked 5000 BC cells (MCF7 or SKBR3 BC cell lines) into 3 mL of healthy blood samples and used ScreenCell Cyto kits to capture those BC cells. Once cells were isolated, a multiplex ICC method was used to stain leukocytes in brown (targeting CD45 common leukocyte marker) and BC cells in red using different markers (Figure 2). This enabled us to check the expression of all those 12 genes at the protein level. We paid special attention to the ratio of positive cells, the intensity of the staining, and the specificity of each targeted protein (absence of expression in leukocytes). Figure 3 demonstrates a representative illustration of ICC staining targeting 12 proteins in the related BC cell lines. This series of experiments revealed that 3 out of 12 markers (MARCKSL1, SLC9A3R1, and RHOD) had a higher expression level and were specific to cancer cells (Table 1).

We then repeated the same experimental procedure, this time with 20 BC cells spiked into healthy blood samples to mimic the patients’ blood condition (the CTCs number is very low, on average between 1 and 100 CTCs per 3 mL of blood). We selected the three proteins with the highest expression levels and demonstrated that MARCKSL1, SLC9A3R1, and RHOD could efficiently identify both BC cell lines (MCF7 and SKBR3) regardless of their rarity in blood samples (Supplementary Figure S2). The combination of these three CTC markers was referred to as the “ScreenCell cocktail”.

To compare the efficacy of conventional EpCAM and CK epithelial markers with our new markers, we evaluated the expression of each marker alone and in combination, i.e., conventional EpCAM + CK cocktail or ScreenCell cocktail, using three markers together.

**Figure 4:**
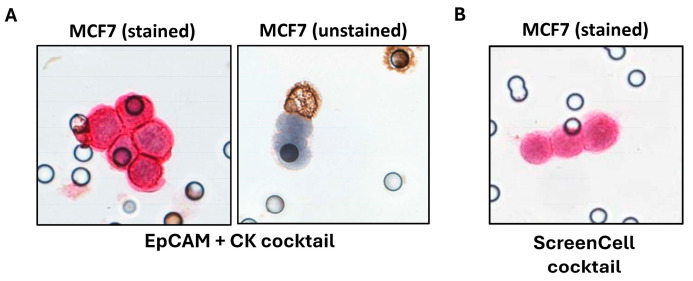
Representative images of ICC staining performed using conventional and ScreenCell cocktails in BC cell lines: 20 MCF7 BC cells were spiked into healthy blood samples, and ScreenCell Cyto technology was used to isolate them. Thereafter, leukocytes are stained in brown using an anti-CD45 antibody, and BC cells are stained in red using (**A**) EpCAM + CK cocktail and (**B**) ScreenCell cocktail. The cell nucleus is stained with hematoxylin. Images are captured at 40×.

While CK alone was able to target 65.60% of the isolated cells, EpCAM labeled only 30.30% of BC cells. The combination of these two markers increased the efficiency of staining up to 69.20%, leaving 30.80% of cells unlabeled (Table 2 and Figure 4A).

We evaluated the same parameter with new markers, either one by one or in combination. While used alone, we observed the following efficiencies: 87.80% for SLC9A3R1, 59.90% for MARCKSL1, and 94.80% for RHOD. The combinations of these markers (MARCKSL1 + SLC9A3R1 + RHOD) when used together (ScreenCell cocktail) led to the staining of 100% of the cancer cells with the uppermost intensity (Table 2 and Figure 4B). Thus, the ScreenCell cocktail showed remarkably higher efficiency in comparison to conventional CK + EpCAM markers (Figure 4).

### 2.4. Clinical Investigations Validated the Efficacy of Novel Markers for CTC Detection

In the next step, we recruited 40 BC patients, all having lymph node invasion, and 18 healthy donors to investigate the clinical validity of our novel markers for identifying CTCs from other cells. ScreenCell Cyto technology was used to isolate CTCs from the blood of these cohorts.

**Figure 5:**
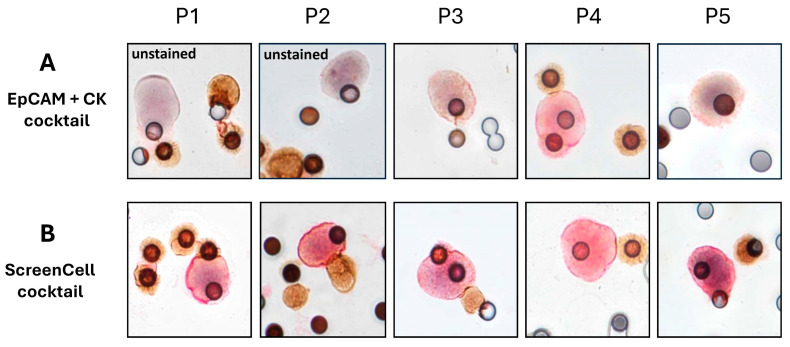
Representative images of ICC staining performed using conventional and ScreenCell cocktails in patients’ samples. Blood samples of the BC patients were processed with ScreenCell Cyto technology to isolate their CTCs. Leukocytes are stained in brown using an anti-CD45 antibody, and CTCs are stained in red using EpCAM + CK cocktail (**A**) or ScreenCell cocktail (**B**) from the same patients. The cell nucleus is stained with hematoxylin. Images are captured at 40×. P: patient.

After the isolation step, atypical cells (ACs) were first selected according to the cytomorphological and immunological criteria described in the method section. Subsequently, the analysis of every selected AC evaluated whether they were positive for the expression of different markers. As anticipated, ICC staining in patients’ samples showed a heterogeneous EpCAM + CK expression, leaving some CD45-ACs (potential CTCs) unstained or weakly stained (Figure 5A). However, when we performed ICC staining using MARCKSL1 + SLC9A3R1 + RHOD markers in the same patients, no ACs remained unstained (Figure 5B).

**Figure 6:**
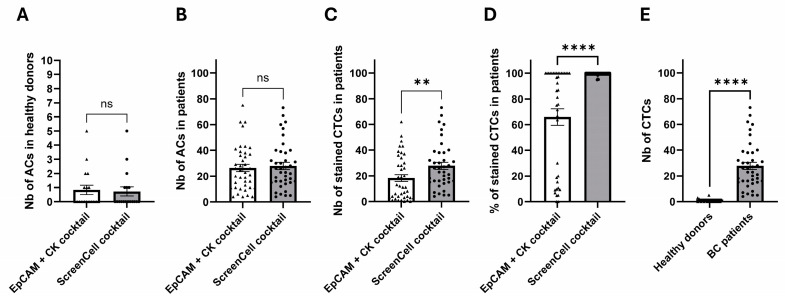
Statistical analysis of CTCs in patients and healthy donor cohorts. (**A**) The number of ACs on different ScreenCell ISs (used for EpCAM + CK or ScreenCell cocktails staining) in healthy donors, (**B**) the number of ACs on different ScreenCell ISs (used for EpCAM + CK or ScreenCell cocktails staining) in BC patients, (**C**) the number of stained CTCs captured by different ScreenCell ISs analyzed by EpCAM + CK or ScreenCell cocktails in BC patients, (**D**) the percentage of stained CTCs after the ICC staining using two different cocktails in patients, (**E**) the number of CTCs after the ICC staining using ScreenCell cocktail in healthy donors and BC patients. Nb: number, ns: non-significant, ** *p*-value = 0.0059, **** *p* < 0.0001 (paired student *t*-test for Figure 6A–D and unpaired student *t*-test for Figure 6E).

We found a comparable number of ACs on different ScreenCell isolation supports (ISs) that were used to assess the expression of the two cocktails. Our results revealed an average of 0.83 and 0.72 ACs in 3 mL of blood in healthy donors (Figure 6A) versus an average of 26.38 and 27.90 ACs in 3 mL of blood for the ISs that were used for staining with EpCAM + CK and the ScreenCell cocktail, respectively (Figure 6B).

After characterization, however, the average number of positively labeled cells was 27.78 cells (99.59%) for the ScreenCell cocktail versus 18.23 cells (65.82%) for the conventional EpCAM + CK cocktail, leaving 34.18% of potential CTCs unstained (Figure 6C,D, and Supplementary Figure S3). Finally, our data showed that there was less than one AC per 3 mL of blood (on average 0.72, median number 0) in healthy donor samples that were all labeled with the ScreenCell cocktail, which is significantly different from the patients’ samples that had, on average, 27.78 CTCs (median number 24.5) in 3 mL of blood (Figure 6E).

These data validate the results obtained with BC cell lines, proving that the ScreenCell cocktail is specific and significantly more sensitive in identifying CTCs.

## 3. Discussion

Most CTC characterization technologies rely either on cytomorphological analyses or on immunological labeling. However, each method has its limitations. Large leukocytes can be confounded with CTCs; some CTCs are small, and some are weakly or not labeled by conventional epithelial antibodies. This leads to mistakes in CTC enumeration, resulting in false-positive or false-negative events. We believe that a concomitant combination of cytomorphological analysis and reliable immunological staining is necessary for the most precise identification of CTCs. For this reason, in our study, we used ICC instead of IF as an immunostaining method.

This study was designed to identify a series of novel markers that could be used to efficiently recognize CTCs. It started with a precise bioinformatics workflow, which was used as a valuable blueprint, continued with in vitro verification using BC cell lines, and finished with clinical validation in BC patients.

Our bioinformatics workflow selected commonly overexpressed genes with membrane expression and high antigenicity among primary tumor cells, BC cell lines, and BC CTCs. BC cell lines were considered as a source of data and the preliminary model in this study, since compared to CTCs, which are extremely rare and hard to maintain in culture, they are accessible and easy to manipulate. This also helped the selection of BC cell lines that express antigens of interest for further investigations. The reason behind applying in silico filtrations (antigenicity and membrane expression) was to increase the chance of recognition by antibodies. Our approach for CTC analysis combines cell morphology assessment with the evaluation of various markers to simplify detection. We use hematoxylin to visualize the cell nucleus in blue, alongside ICC staining for different antigens in red and brown. Utilizing membrane markers enhances the visibility of nuclei and allows for more accurate marker evaluation. Indeed, membrane labeling of the antigens does not interfere with an accurate cytomorphological characterization that requires intact conservation of intracellular features. In contrast, cytoplasmic markers can obscure details and make differentiation challenging. These conditions also provide an opportunity for further studies focused on CTC eradication either by the education of the immune system or by specifically designed antibodies.

Upon in silico selection of 12 potential markers, we used MCF7 and SKBR3 BC cell lines to verify their protein expression level. Those cells were spiked into healthy blood samples, and ScreenCell technology was used to capture them. ScreenCell is a cost-effective, simple-to-use, and sensitive technology that isolates CTCs based on their size [29,34]. We have already demonstrated that the ScreenCell recovery rate is 91.6% for 10 cells and 86.6% for as few as 5 cells [30]. This permitted us to rigorously compare the expression of our selected markers with EpCAM and CK individually or combined. This essential in vitro step enabled us to keep only SLC9A3R1, MARCKSL1, and RHOD as the most potent markers.

To clinically validate the performance of our novel markers, we then recruited a cohort of 40 LN-invaded BC patients and 18 healthy donors. The initial cytomorphological and immunological analyses revealed that all patients’ samples contained ACs (27.14 on average), while healthy donors had significantly lower events (0.77 on average). We then performed downstream ICC staining analysis to compare the sensitivity of EpCAM and CK conventional markers to our novel markers. Indeed, the ACs that are labeled with EpCAM and CK markers are commonly identified as CTCs. However, according to our and others’ results, some ACs (CD45 negative potential CTCs) remain unstained using these canonical markers, leading to an underestimated CTC evaluation [24,25,26,27]. However, while using SLC9A3R1, MARCKSL1, or RHOD markers individually, we already observed a significant improvement in detecting CTCs compared to EpCAM or CK markers. A combination of all those markers in a cocktail maximized the CTC detection up to 99.59%. This is 33.77% more efficient compared to conventional markers. This is not unspecific staining: first, we did not observe the same results in healthy donor blood samples, where we found less than 1 CTC in 3 mL of blood, and second, the initial number of ACs was comparable (an average of 26.38 and 27.90 ACs) for the ISs that were used to assess the two cocktails.

In our study, the initial step to facilitate CTC detection involved CD45 staining to eliminate leukocytes. While the ScreenCell markers validated many of the cells identified as CTCs based on their morphology and absence of CD45 expression, we found that relying solely on negative markers or morphological parameters alone can be insufficient. Without using positive markers, the thorough characterization required for each cell sample can take an expert pathologist a considerable time per patient. To streamline the process and improve CTC detection, we employ a combination of positive CTC markers. This approach significantly accelerates characterization compared to using negative markers or morphology alone. The positive markers help ensure the identification of all relevant CTCs, including those that may fall outside the typical 20-micrometer size range.

Our data proves the high probability of finding CTCs in LN-invaded cancer stages and the efficiency of ScreenCell technology in isolating them. This is in accordance with the pooled analysis by Janni et al., showing that the presence of CTCs was associated with higher histological grade, LN involvement, and tumor size [17].

While using EpCAM and CK markers, we sometimes observed a slight staining with the DAB reagent (Figure 5), which is used to reveal CD45 labeling. This phenomenon could be either due to a background noise effect of the DAB reagent or a low expression of CD45 molecules on some CTCs. This latter hypothesis has recently been validated by Yang et al., demonstrating that the CD45 labeling on this subpopulation of CTCs was attributed to the presence of leucocyte-originated exosomes on the cytoplasm of these cells. The presence of CD45+ CTCs is linked to a malignant phenotype with higher metastatic potential compared to CD45- CTCs [35]. While using the ScreenCell cocktail, we did not see this CD45+ CTC population, showing that this cocktail can efficiently identify all CTCs regardless of their biological status.

Indeed, previous studies demonstrated the important roles of MARCKSL1, SLC9A3R1, and RHOD. MARCKSL1 (myristoylated alanine-rich C-kinase substrate-like 1) plays a role in cytoskeletal regulation, formation of adhesion junctions, protein kinase C signaling, and calmodulin signaling. It was shown that its expression is associated with poor prognosis in ovarian cancer [36] and in inflammatory BC [37,38], promoting the proliferation, migration, and invasion of lung cancer [39]. Interestingly, MARCKS inhibition was reported to reduce the migration and invasion of prostate cancer cells [40].

SLC9A3R1 (sodium-hydrogen antiporter 3 regulator 1) is a regulator of sodium-hydrogen antiporter 3. It is a scaffold protein that links ezrin, moesin, and radixin family members to plasma membrane proteins, helping to connect them to the actin cytoskeleton to control their surface expression [41,42]. Controversial data exist about the role of this protein in cancer. In prostate cancer, for example, the upregulation of SLC9A3R1 has been associated with the carcinogenic potential of this type of cancer [43]. In contrast, SLC9A3R1 has been shown to suppress proliferation in BC via altering the expression of phosphatase and tensin homolog, as well as by interfering with the transduction of growth signals brought on by the EGFR and PDGFR [44,45]. Another study showed that SLC9A3R1 (NHERF1) differentially regulates the expression of two phenotypic programs through its PDZ domains. The PDZ1 domain promotes bone metastases by increasing podosome nucleation, motility, neo-angiogenesis, vasculogenic mimicry, and osteoclastogenesis in the absence of increased growth or invasion, whereas the PDZ2 domain promotes visceral metastases through increased invadopodia-dependent invasion and anchorage-independent growth, as well as by inhibiting apoptosis [46].

RHOD (Ras homolog gene family, member D) is a small signaling G protein and a member of the Rho family of GTPases, which is involved in endosome dynamics and recognition of the actin cytoskeleton and regulation of cell migration. Interestingly, previous studies connected RHOD expression to tumorogenesis, higher tumor invasion capacity, and shorter survival [47]. Additionally, some studies correlated RHOD expression to a higher BC risk, making it a very interesting target for cancer investigations [48].

The combination of these three markers in a cocktail for identifying CTCs paves the way for investigating a wide range of cellular functions, which should be explored in future studies. Additionally, while these markers allowed for the detection of ACs in samples, we did not perform molecular characterization to validate the cancerous origin of the cells detected. Further molecular investigation would be needed to confirm whether this approach can reliably identify cancer cells versus other non-cancerous epithelial cells that may be present in the blood. Another limitation of this study is the restricted use of MCF7 (HR+ HER2−) and SKBR3 (HR− HER2+) BC cell lines for in vitro validations. Indeed, these two commonly used cell lines do not represent the TNBC condition, which is the most aggressive subpopulation of BC. In our current study, only 10% of the cohort was TNBC. In our future studies, we will include more TNBC patients to have a better understanding of this aggressive population. The last limitation concerns the cohort of healthy donors, whose differences in ethnicity and lack of detailed profiles, such as their exact age due to confidentiality, might introduce bias into the results. In future clinical trials, we plan to focus on recruiting individuals with more comparable characteristics.

## 4. Methods

### 4.1. Patients, Cohort Description, and Healthy Blood Samples

The blood samples of 40 LN-invaded BC patients and 18 healthy donors were analyzed in this study. The blood samples of BC patients were collected in the Kahia laboratory in Oran, Algeria. This clinical study was approved locally by the ethical committee of the Faculty of Medicine of the University of Oran 1, Oran, Algeria (Ministry of Higher Education and Scientific Research) under the authorization number 02/CED/FACMED/2023. It was further approved at the national level by the Algerian Ministry of Industry and Pharmaceutical Production, Directorate of Production, Industrial Development, Export Promotion, and Research under the registration number 013/LAK/OBS/DM-DIV/2023. The international biological transfer authorization was delivered by the Algerian Ministry of Industry and Pharmaceutical Production under the following number: 013/LAK/A-TR/DM-DIV/2023. All patients signed the consent form before inclusion in the study.

Healthy blood samples were received from the French Blood Establishment (EFS) located at La Pitié Salpêtrière Hospital, Paris, France, under the authorization number 2022-2026-042 CCPSL.

Cohort description:

This study involved 40 BC patients who presented with one or more invaded LNs at diagnosis. At the time of diagnosis, 21 out of 40 patients were under 50 years of age. Tumor grading revealed that 14 patients (35%) had grade 3 tumors, while the remainder had grade 2 tumors. The distribution of tumor molecular subtypes included 14 patients (35%) with hormone receptor-positive (HR+) and human epidermal growth factor receptor 2 negative (HER2−) tumors, 11 patients (27.5%) with HR + HER2+ tumors, 8 patients (20%) with HER2+ tumors, and 4 patients (10%) with triple-negative breast cancer (TNBC). Immunohistochemistry (IHC) could not be performed on three patients. The predominant histological type was infiltrating ductal carcinoma (IDC), observed in 33 patients (82.5%), while infiltrating lobular carcinoma (ILC) was present in 7 patients (17.5%). Additionally, 16 of the 40 patients had tumors of size 4, and 65% of the cohort exhibited a stromal fibro-inflammatory reaction within their tumor microenvironment. Furthermore, 9 women (22.5%) reported a family history of breast cancer.

### 4.2. Preparation of Different Experimental Conditions Using Blood Samples

A maximum of two K2-EDTA tubes of 9 mL were drawn per patient. The blood was filtered locally in a devoted clinical lab, and between 3 and 5 CTC ISs were prepared for each patient using ScreenCell Cyto kits (ScreenCell, Paris, France) (5 filtrations of 3 mL of blood for the first 11 patients and 3 filtrations of 3 mL of blood for the rest of the patients) (Supplementary Figure S4). In addition to CD45 staining that was performed for all conditions, for the first 11 patients, the first IS was used to stain the isolated CTCs with EpCAM + CK (conventional cocktail). The next 3 ISs were dedicated to assessing the expression of 3 new markers individually (MARCKSL1, SLC9A3R1, RHOD). The last IS was used to stain the cells with a combination of 3 new markers (ScreenCell cocktail) (Supplementary Figure S4A). For patients 12 to 40, 1 IS was used for the conventional cocktail, 1 IS for the ScreenCell cocktail, and the last one was saved for further or retrospective analysis (Supplementary Figure S4B).

Concerning the healthy donors, 1 K2-EDTA blood collection tube (9 mL of blood in total) was filtered with ScreenCell Cyto kits to generate 3 IS/donor; 1 IS was immunostained with the EpCAM + CK (conventional cocktail), and 1 IS was stained with the ScreenCell cocktail. The last IS was saved for further or retrospective analysis (Supplementary Figure S4B).

### 4.3. Public Omics Datasets

#### 4.3.1. Transcriptome Dataset of Cancer Cell Line Encyclopedia (CCLE) GSE36133

The Gene Expression Omnibus dataset GSE36133 was used for the transcriptome expression analysis performed by Affymetrix Human Genome U133 Plus 2.0 Array technologies. The whole transcriptome experiments were processed on RNA from the cancer cell line collection [49]. The normalized transcriptome matrix was downloaded and annotated with the GEO platform GPL15308 (Brainarray Version 15.0.0, HGU133Plus2_Hs_ENTREZG). An annotated matrix was filtered to study the heterogeneity of BC cell lines.

#### 4.3.2. Transcriptome Dataset of Primary Breast Tumors and Breast-Adjacent Tissues GSE93601

Supervised transcriptome analysis was performed on the GEO dataset GSE93601 directly online through the GEO2R NCBI web application to elucidate differentially expressed genes found between primary breast tumor samples and breast-adjacent tissue samples [50]. The linear model for the microarray (LIMMA) algorithm [51] was run with default parameters between selected groups of samples: primary breast tumors (BT) versus adjacent breast tissue samples (BNT). Gene significance was retained when the False Discovery Rate adjusted *p*-values were <0.05.

#### 4.3.3. Single-Cell Transcriptome Dataset of Circulating Tumor Cells of Breast Cancer GSE109761

FPKM normalized matrix of single-cell transcriptome BC CTCs from the GEO dataset GSE109761 [52] was downloaded from the website of the ctcRbase database [53]. From the matrix, the CTC cluster information was removed to keep only the single-cell transcriptome performed on unique cells.

#### 4.3.4. Breast Cancer TCGA RNA-Sequencing Dataset Associated with Clinical Data

As an independent testing cohort, the TCGA BC dataset consisted of 817 patients/samples [54]. The RNA-sequencing V2 Z-scores (diploid) matrix was downloaded from the Cbioportal web application [55] with associated clinical data. Respective tables were imported into R software as data frames to process downstream survival analysis following the RNA-sequencing expression of selected markers.

#### 4.3.5. Bioinformatics Analysis

The general workflow of bioinformatics analyses performed during this study is described in a diagram in Figure 1. Bioinformatics analyses were performed in the R software environment version 4.1.0. R-package dplyr version 1.0.7 was used to perform a join between data tables. A Venn diagram between gene lists was performed with the Venny web application, which is available at the following address: https://bioinfogp.cnb.csic.es/tools/venny/ (accessed on 30 November 2021). The graphical output of the principal component analysis was performed with the autopilot function of the ggfortify R package version 0.4.13. Transcriptome expression heatmaps were drawn with the heatmap R package version 1.0.12. The selection of plasma membrane-expressed molecules among transcriptome expression profiles was completed by querying the Gene Ontology Cell Compartment database [56]. Disease functional enrichment of the gene list was performed with the ToppGene web application suite [57] run on the DisGeNet database [58].

#### 4.3.6. Selection of Highly Variable Expressed Genes in Transcriptome by Parametric and Unsupervised Approaches

An unsupervised approach was used to select higher variables and genes expressed through a transcriptome expression dataset, and a specific R function was developed. This function, named “overmean”, is available freely at the following address: https://github.com/cdesterke/overmean (accessed on 30 November 2021). This function uses as an input parameter a transcriptome expression dataset with unique identifiers as “row.names” and outputs a subset dataset with selected genes in rows. Selected genes were considered expressed because they had an expression over the mean of the array and also variable because they had a between-sample variance over that of the arrays in the dataset.

#### 4.3.7. Protein Sequence Antigenicity Estimation

The complete proteome sequence FASTA file of Homo sapiens (assembly GRCh38.p13) was downloaded from the NCBI website: https://www.ncbi.nlm.nih.gov/ (accessed on 30 November 2021). The human proteome FASTA sequence file was a subset of the multi-FASTA file of the proteins corresponding to the genes selected (144 genes, Figure 1), with SeqTK v0.16.1 installed under the Ubuntu 20.04 LTS operating system (https://github.com/lh3/seqtk, accessed on 30 November 2021). The corresponding output subset multi-FASTA file was processed for antigenicity quantification with the Vaxijen 2.0 algorithm (https://ddg-pharmfac.net/vaxijen/VaxiJen/VaxiJen.html, accessed on 30 November 2021).

#### 4.3.8. In Silico Sorting

To refine the list of 50 genes retained by bioinformatics analysis, we performed a secondary in silico analysis using bioinformatics databases such as The Human Protein Atlas and UniProt. For this analysis, we selected our targets based on the following criteria: (I) selection of proteins with confirmed membrane localization in both databases, (II) exclusion of proteins expressed by immune cells, and (III) selection of proteins with higher expression in primary BC tumors, as determined by the IHC method. This approach enabled us to identify 12 potential markers. Additionally, analysis of the public RNAseq data highlighted the MCF7 and SKBR3 BC cell lines as suitable models to validate their expression levels (Supplementary Figure S1).

#### 4.3.9. Cell Line Preparation and Culture

MCF7 (HR+ HER2−) and SKBR3 (HR− HER2+) cancer cell lines were used in this study. Both cell lines were obtained from the French National Institute of Health and Medical Research (Inserm) U1197, located at Paul Brousse Hospital, Villejuif, France. MCF7 (passages 5 to 12) and SKBR3 (passages 5 to 10) were cultured in DMEM (Gibco, Thermofisher, Waltham, MA, USA) and McCoy’s (ATCC, Manassas, VA, USA), supplemented with 10% fetal bovine serum (FBS) (Pan biotech, Aidenbach, Germany) and 1% penicillin-streptomycin (PS) antibiotics (Gibco, Thermofisher, Waltham, MA, USA). Cells were detached using trypsin (Gibco, Thermofisher, Waltham, MA, USA). Then, 5000 or 20 cells were spiked into 3 mL of healthy blood that was collected in K2-EDTA 9 mL standard blood collection tubes.

#### 4.3.10. ScreenCell Technology

The whole blood was collected in K2-EDTA tubes and processed within 4 h after collection. Blood samples were processed according to ScreenCell’s instructions using the ScreenCell Cyto kit (Screencell, Paris, France). Briefly, a volume of 3 mL of blood samples was diluted with 4 mL FC2 dilution buffer to allow red blood cell (RBC) lysis and the preservation of the nucleated cells during 8 min of incubation time [29]. At the end of the enrichment step, the IS (the porous membrane located in the intermediate part of the ScreenCell Cyto device) was cleaned from blood waste using 1.6 mL PBS 1X and released, air-dried overnight, and colored with RAL555 (Cat# 720-0351, VWR, International, Radnor, PA, USA) for cytomorphological studies. The IS was then covered with an 8 mm circular glass coverslip on a glass slide without a mounting medium. The characterization and enumeration of CTCs were performed by an experienced pathologist (JW), blinded to the histological diagnosis, using a NIKON C_TEP3 (ref: 553494) fluorescence microscope integrated with a cooled CCD camera system, Nikon DS-FI3, and NIS_Elements BR version 5.42.03 imaging software (NIKON, Paris, France). Captured cells displaying the following features, (1) absence of intense and homogeneous CD45 common leukocyte marker expression, (2) cells larger than the average size of leucocytes, (3) irregular nuclear outline, and (4) high nuclear/cytoplasmic ratio (>0.75), were initially classified as atypical cells (ACs) in comparison to leukocytes. If ACs were positive for EpCAM and CK markers, they were considered CTCs, and if they were negative for these markers, they were considered potential CTCs. After the validation steps, ACs were also considered CTCs if they were positive for MARCKSL1, SLC9A3R1, and RHOD markers.

#### 4.3.11. Immunocytochemistry

ScrennCell ISs were first hydrated with tris-buffered saline (TBS) (Dakocytomation, Glostrup, Denmark) containing 0.05% Tween 20. If necessary, the cells were permeabilized with TBS-Triton 0.2%. The antigens were retrieved with target retrieval solution S1699 diluted 1X (Dako, Glostrup, Denmark) at 96 °C for 20 min and rinsed with TBS-Tween 0.05%. Manual DAB/RED ICC double staining was carried out using the chromoplex 1 detection kit (Leica Biosystems, Nanterre, France). Isolated cells were treated for 5 min at room temperature with a peroxidase block solution (LeicaBiosystems, Nanterre, France).

Primary antibody staining was performed for 30 min at room temperature and 30 min at 4 °C with the following anti-human antibodies: antibodies produced in rabbit: anti-Rhod AV42414, 1/50 dilution, anti-SLC9A3R1 HPA009672, 1/50 dilution, anti-MARCKSL1 HPA030528, 1/250 dilution, anti-EpCAM HPA026761, 1/50 dilution, all from Sigma Aldrich, Saint-Louis, United States, and anti-cytokeratin 8, SU0338 from Invitrogen, Massachusetts, United States, 1/100 dilution. Mouse primary anti-CD45 PA0042 (Leica Biosystems, Nanterre, France, ready to use) was used for leukocyte staining. When validating the assay with 5000 spiked cancer cells, we used the highest recommended antibody concentration, but increased it fourfold when testing with only 20 spiked cells to overcome unstained cells. This approach was applied consistently to all markers.

A post-primary polymer mHRP (Poly-HRP anti-mouse, Leica Biosystems, Nanterre, France) was then applied for 8 min at room temperature, followed by a rinsing step with TBS-Tween 0.05%, and the addition of polymer rAP (Poly-AP anti-rabbit, Leica Biosystems, Nanterre, France) for 20 min at room temperature. Finally, chromogenic staining using DAB-RED detection according to Leica Biosystems protocol and a counter-staining with Hematoxylin (Epredia, Kalamazoo, MI, USA) for 5 min at room temperature allowed the revelation of the antigen detection. After a final wash with distilled water, the ScreenCell^®^ Cyto ISs were mounted on a glass slide with the Faramount mounting medium (Dako, Glostrup, Denmark) and covered with an 8 mm diameter coverslip.

#### 4.3.12. Statistical Analysis

GraphPad Prism 10 was used for statistical analysis. Descriptive statistics were used to calculate the mean values and the standard error of the mean (SEM). Paired student *t*-test and unpaired student *t*-test were performed for *p*-value generation depending on the experimental conditions.

## 5. Conclusions

This comprehensive study was based on bioinformatics, in vitro experiments using BC cell lines, completed by a final validation in 40 LN-invaded BC patients and 18 healthy donors. Our results validated a trio signature of markers that could be used along with cytomorphological characterization to more efficiently identify CTCs compared to conventional EpCAM and CK epithelial markers. Additionally, it validates the clinical efficacy of ScreenCell technology in successfully isolating CTCs from LN-invaded BC patients.

## Figures and Tables

**Table 1:** List of 12 identified genes and their protein expression in breast cancer cell lines and leukocytes. The gray background corresponds to those proteins with a higher expression level.

Protein	Breast Cancer Cell Line	Expression Profile	Expression by Leukocytes
CD55	SKBR3	Low	No
LSR	MCF7	Medium	No
MARCKSL1	MCF7	Medium/High	No
GPC1	SKBR3	Low	No
SLC9A3R1	MCF7	High	No
CXADR	SKBR3	Low	No
SHROOM3	SKBR3	Low	No
MUC1	MCF7	Low/Medium	No
PPP1R16A	MCF7	Low	No
ATP1B1	SKBR3	Medium	No
RHOD	MCF7	High	No
SYTL2	MCF7	Medium/High	Yes

**Table 2:** Expression of different markers, alone or in combination, using 20 MCF7 cells spiked into healthy blood samples. ScreenCell cocktail = MARCKSL1 + SLC9A3R1 + RHOD markers. N = 4 for EpCAM and CK conditions, and n = 3 for the rest of the conditions. Low and High refer to the expression levels confirmed by the different experimenters. Positive: the addition of low-expression and high-expression intensity conditions.

	CK	EPCAM	CK + EPCAM COCKTAIL	SLC9A3R1	MARCKSL1	RHOD	SCREENCELL COCKTAIL
**NEGATIVE**	34.40%	69.70%	30.80%	11.50%	40%	5.12%	0%
**LOW**	16.40%	22.70%	25%	37.70%	13.30%	41%	1.90%
**HIGH**	49.20%	7.60%	44.20%	50.10%	46.60%	53.80%	98.10%
**POSITIVE**	**65.60%**	**30.30%**	**69.20%**	**87.80%**	**59.90%**	**94.80%**	**100%**

## Data Availability

The datasets used and/or analyzed during the current study are available from the corresponding author on reasonable request.

## Supplementary Materials

The following supporting information can be downloaded at: https://www.mdpi.com/article/10.3390/ijms26104714/s1.

## Abbreviations

BC	breast cancer
CK	cytokeratin
CTC	circulating tumor cell
DEG	differentially expressed gene
dPCR	digital polymerase chain reaction
EMT	epithelial to mesenchymal transition
EpCAM	epithelial cell adhesion molecule
HER2	human epidermal growth factor receptor 2
HR	hormone receptor
ICC	immunocytochemistry
IDC	infiltrating ductal carcinoma
IF	immunofluorescence
IHC	immunohistochemistry
ILC	infiltrating lobular carcinoma
IS	isolation support
NGS	next-generation sequencing
OS	overall survival
PDL1	programmed death-ligand 1
PFS	progression-free survival
RBC	red blood cell
TNBC	triple-negative breast cancer
WHO	World Health Organization
