# Supplementary material for: Optimizing Detection of Circulating Tumor Cells in Breast Cancer: Unveiling New Markers for Clinical Applications
Source: Int J Mol Sci. 2025 May 14;26(10):4714. doi: 10.3390/ijms26104714 (PMC12111556; doi:10.3390/ijms26104714)
Supplement: Supplementary file 1 [file ijms-26-04714-s001.zip › ijms-3568314-supplementary.pdf]

A

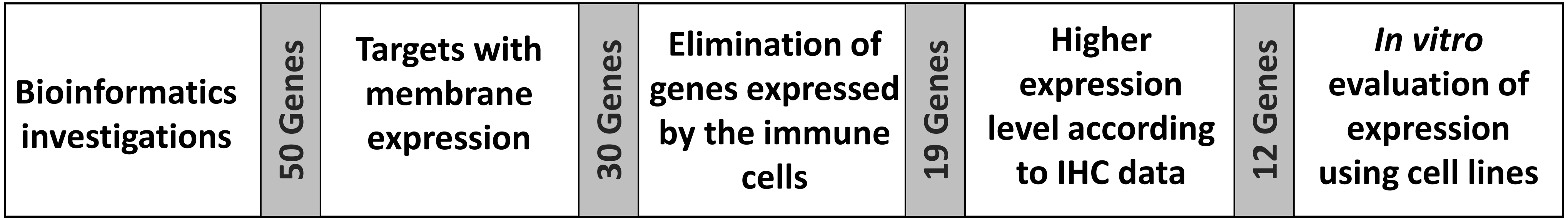

B

Genes	Breast cancer cell line
CD55	SKBR3
LSR	MCF7
MARCKSL1	MCF7
GPC1	SKBR3
SLC9A3R1	MCF7
CXADR	SKBR3
SHROOM3	SKBR3
MUC1	MCF7
PPP1R16A	MCF7
ATP1B1	SKBR3
RHOD	MCF7
SYTL2	MCF7

Supplementary Figure S1. In silico sorting strategy: (A) Workflow of gene filtration using Protein Atlas and UniProt databases. (B) The results of the analysis of public RNAseq data highlighted the MCF7 and SKBR3 BC cell lines as suitable models to validate the expression levels of our selected targets.

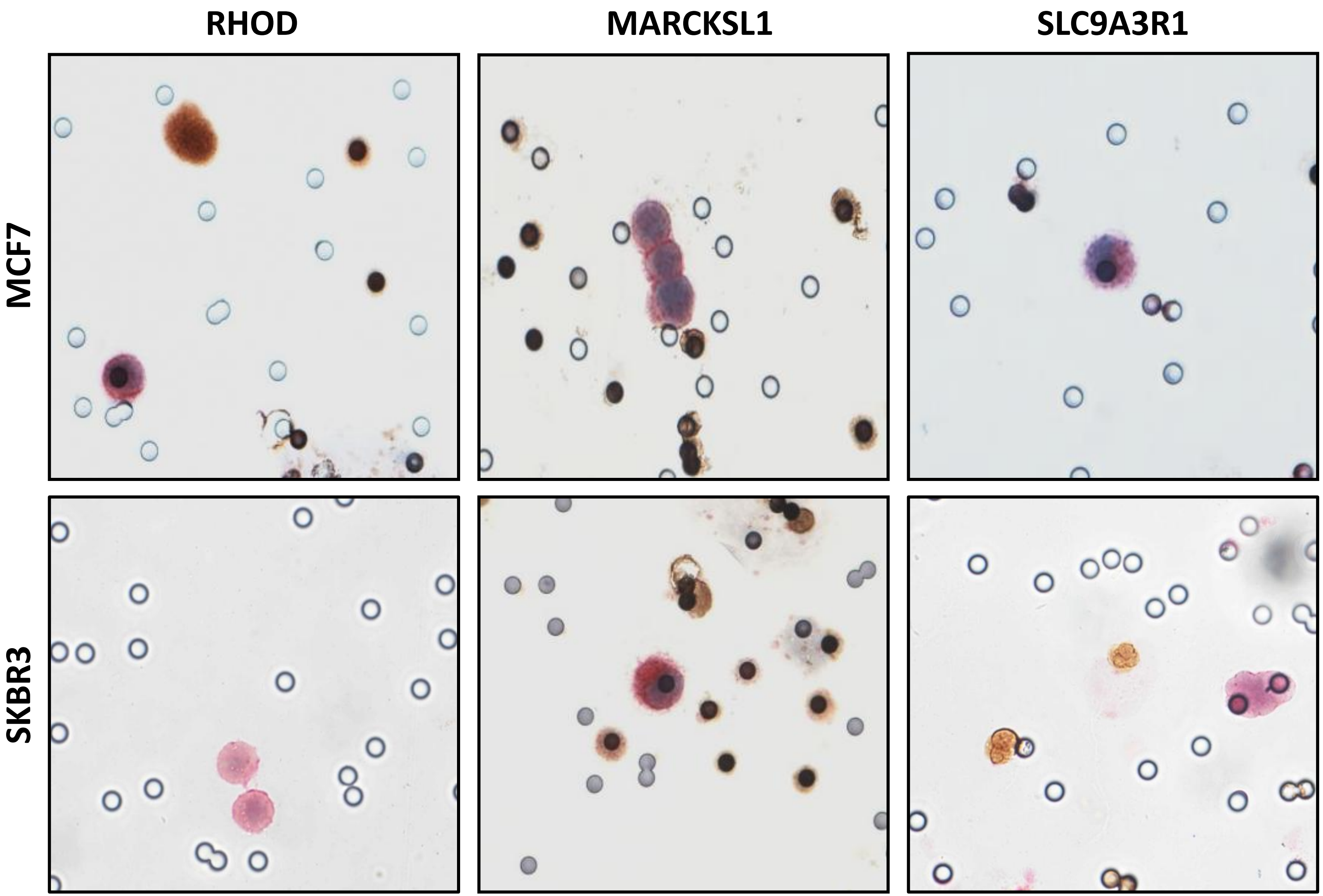

Supplementary Figure S2. Representative images of ICC immunostaining in two BC cell lines. We selected 3 proteins with higher expression levels and demonstrated that SLC9A3R1, MARCKSL1, and RHOD are positive in both BC cell lines (MCF7 and SKBR3). Leukocytes are stained in brown using an anti-CD45 antibody, and BC cells are stained in red using each marker of interest. The cell nucleus is stained with hematoxylin. Images are captured at 40X.

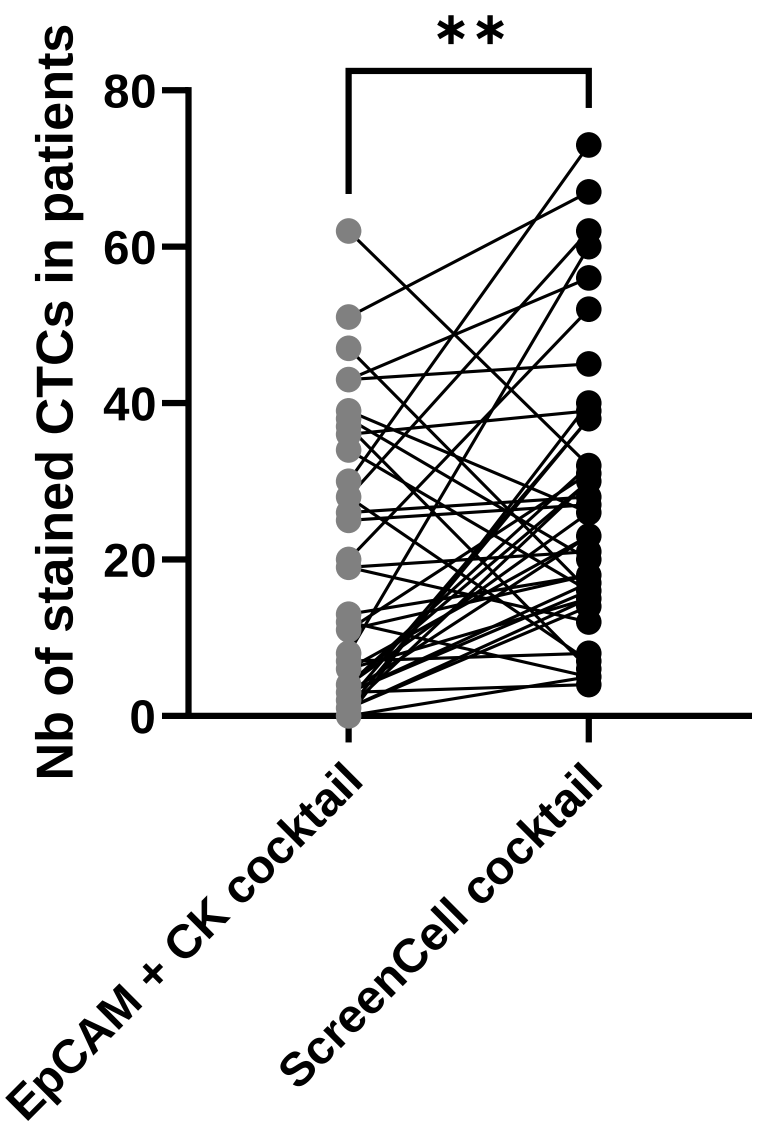

Supplementary Figure S3. Statistical analysis of CTCs in the patient's cohort. A comparison between the number of stained CTCs in each patient characterized by different cocktails (EpCAM + CK or ScreenCell cocktail). Nb: number, ** p-value = 0.0059 (paired student t-test).

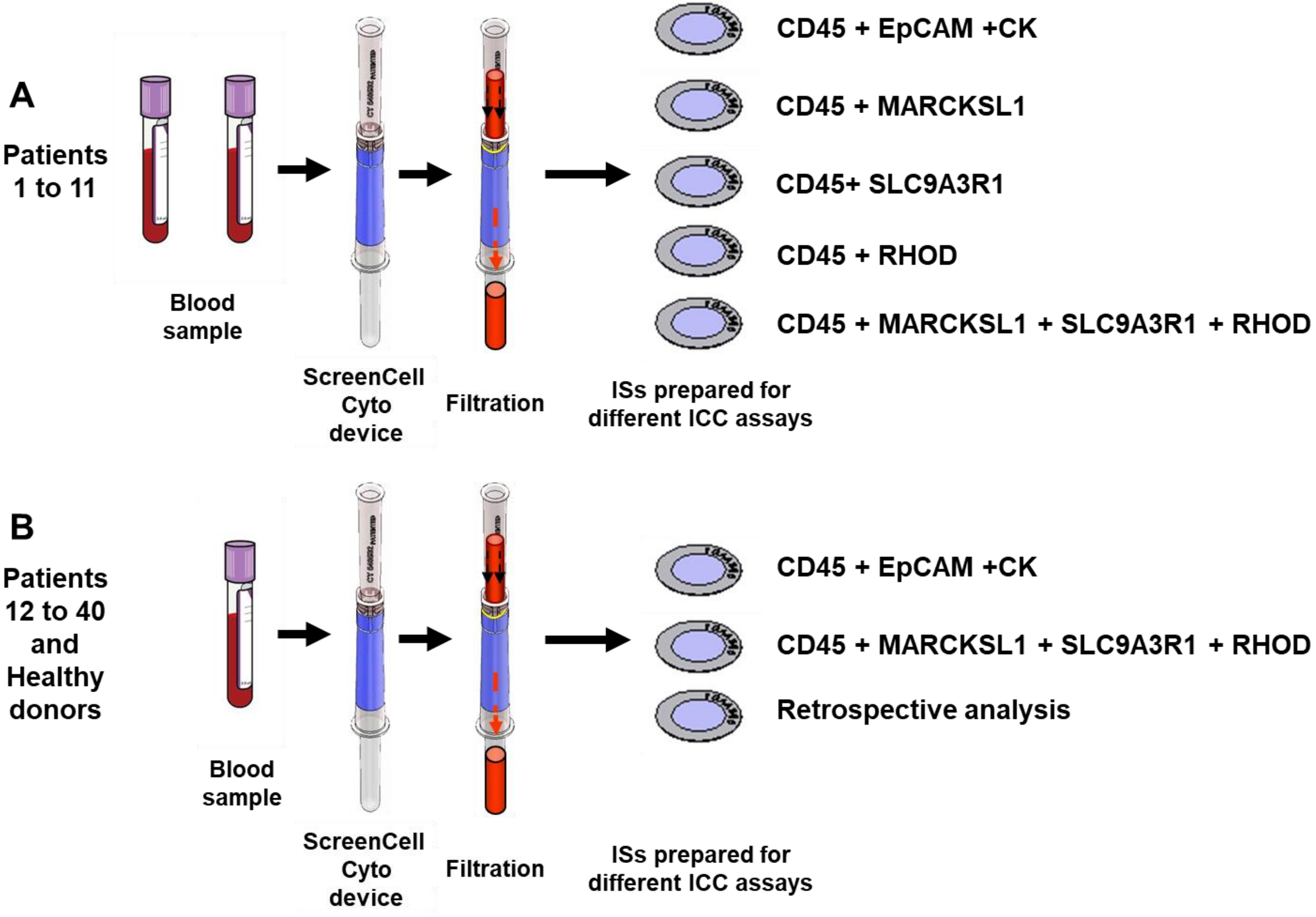

Supplementary Figure S4. Preparation of different experimental conditions using blood samples. ScreenCell Cyto technology was used to isolate CTCs from the blood samples. (A) For patients 1 to 11, 2 K2-EDTA blood collection tubes were used to generate 5 ISs to assess the expression of different markers alone or in combination. (B) For patients 12 to 40 and 18 healthy donors, 1 K2-EDTA blood collection tube was used to generate 3 ISs. 2 ISs were used to assess the expression of EpCAM + CK conventional cocktail and ScreenCell cocktail (MARCKSL1 + SLC9A3R1 + RHOD), and the last one was kept for retrospective analysis. ISs: isolation supports.
